# Exercise Prior to Lower Extremity Peripheral Artery Disease Improves Endurance Capacity and Hindlimb Blood Flow by Inhibiting Muscle Inflammation

**DOI:** 10.3389/fcvm.2021.706491

**Published:** 2021-08-04

**Authors:** Maxime Pellegrin, Karima Bouzourène, Lucia Mazzolai

**Affiliations:** Division of Angiology, Heart and Vessel Department, University Hospital of Lausanne (CHUV), Lausanne, Switzerland

**Keywords:** exercise, lower extremity peripheral artery disease, prevention, skeletal muscle, arteriogenesis, regeneration, inflammation, monocyte/macrophage phenotype

## Abstract

Lower extremity peripheral artery disease (PAD) is associated with functional decline. Physical exercise has been proven to be an effective therapeutic strategy for PAD; however the effect of exercise initiated before PAD remains unknown. Here, we investigated the preventive effects of exercise on endurance capacity, hindlimb perfusion, and on polarization profile of circulating monocytes and limb muscle macrophages. ApoE^−/−^ mice were subjected to 5-week running wheel exercise or remained sedentary before induction of hindlimb ischemia. The two groups were thereafter kept sedentary. Exercised mice prior to PAD showed higher exhaustive treadmill running distance and time than sedentary mice. Preventive exercise also increased perfusion, arteriole density, and muscle regeneration in the ischemic hindlimb. Moreover, preventive exercise prevented ischemia-induced increased gene expression of pro-inflammatory M1 macrophages markers and cytokines in the ischemic muscle, while no changes were observed for anti-inflammatory M2 macrophage markers. Flow cytometry analysis showed that the proportion of circulating pro-inflammatory monocyte subtype decreased whereas that of anti-inflammatory monocytes increased with preventive exercise. Overall, we show that exercise initiated before PAD improves endurance performance and hindlimb perfusion in mice probably *via* inhibition of M1 macrophage polarization and inflammation in the ischemic muscle. Our study provides experimental evidence for a role of regular exercise in primary prevention of PAD.

## Introduction

Peripheral artery disease of lower extremities (PAD) due to occlusive atherosclerotic plaques in lower extremity arteries is a major cause of disability and mobility loss. Intermittent claudication (IC) is considered the most common symptomatic presentation of PAD, characterized by ischemic muscle pain during physical activity due to inadequate blood flow (1). Consequently, IC leads to functional impairment and decline. Improving functional performance and/or preventing functional decline are therefore major therapeutic goals for patients with PAD (1).

Physical activity is associated with a reduced risk of developing chronic cardiovascular disorders, including coronary artery disease, heart failure, and stroke (2, 3). The beneficial effects of exercise training for the secondary prevention of cardiovascular disease is likewise widely demonstrated (2, 3). Exercise training, and in particular regular walking exercise, has also a central role in PAD management based on considerable evidence demonstrating that it provides important benefits in terms of walking performance in PAD patients (4–8). We and others have also recently reported improved running capacity following exercise training in a mouse model of PAD/hindlimb ischemia (9, 10). Although the therapeutic effectiveness of exercise interventions for individuals with established PAD has been widely documented, it remains unknown, whether exercise initiated before the onset of disease could also be a valuable strategy for alleviating disease severity.

Monocytes and macrophages are important components of the innate immune system playing a key role in arteriogenesis and tissue/muscle repair following ischemia. Macrophages undergo phenotype change according to specific environmental cues. Macrophages first infiltrating the ischemic muscle are polarized to the pro-inflammatory M1 (classically activated) phenotype (11, 12). After predominantly early infiltration of M1 macrophages, a shift toward the anti-inflammatory M2 (alternatively activated) phenotype appears in later stage post-ischemia, promoting collateral growth and enhance blood flow recovery (11, 12). Therefore, therapeutic interventions aiming at modulating the phenotype of macrophages in the ischemic muscle has been proved to be effective in improving perfusion in experimental PAD (13–17).

Exercise has been shown to have anti-inflammatory properties and the capacity to influence monocytes/macrophage phenotype in the circulation and/or in different tissues including lower extremity skeletal muscle both in human (18, 19) and animal models (20, 21). However, to our best knowledge, whether exercise is effective in modulating the polarization of monocyte/macrophage in the circulation and ischemic muscle tissue in PAD has not been previously investigated.

The present study therefore investigated the effect of exercise prior to PAD on functional capacity, limb perfusion and arteriogenesis, and on polarization profile of circulating monocytes and limb muscle macrophages in Apolipoprotein E-deficient (ApoE^−/−^) mice.

## Methods

### Animal Protocol

In this study, a total of 38 C57BL/6 ApoE^−/−^ male mice were used. Mice were originally obtained from Charles River Laboratories (L'arbresle, France) and subsequently bred and maintained under conventional housing conditions at the animal facility of the University of Lausanne and University Hospital of Lausanne. We used hypercholesterolemic ApoE^−/−^ mice to closely mimic the human disease, as PAD patients commonly have hypercholesterolemia. Mice were given regular rodent chow and water ad libitum.

At 7-8 weeks of age, mice were divided into two groups: the first one underwent voluntary exercise training (EXE) for 5 weeks, while the second group remained sedentary (SED) during the 5-week period. Mice in the EXE group had free access to a 12-cm diameter running wheel (Intellibio, Seichamps, France) 24 h a day, 7 days a week. Number of revolutions was recorded daily.

At the end of the exercise or sedentary period, mice underwent unilateral iliac artery ligation to induce PAD and hindlimb ischemia as described previously (10, 14). After arterial ligation, mice were kept sedentary for either 5 weeks (first set of experiments; *n* = 22 mice) or 1 week (second set of experiments; *n* = 16 mice) before euthanasia.

### Exhaustive Treadmill Running Test

Maximal running distance and maximal running time were assessed using an incremental forced running test on a motor-driven treadmill (Columbus Instruments, USA) (14). The test started by running at 5 m/min for 5 min (warm-up), followed by speed increments (2 m/min every 3 min). An electrical grid (0.2 mA, 3 Hz) was placed at the back of the treadmill to provide motivation. The test was stopped when mice were exhausted, i.e., when they remained on the electrical grid for 5 s. The test was repeated twice for each time point, and results were averaged. Mice were allowed to acclimate to the treadmill for 1 week before the first test (10-min running at 5 m/min).

### Laser Doppler Hindlimb Perfusion Measurement

Tissue perfusion of ischemic and contralateral non-ischemic hindlimbs was evaluated using a laser Doppler Imager (Moor Instruments Ltd, Axminster, England) in anesthetized mice as described earlier (10, 14). In brief, five consecutive plantar foot images of hindlimbs were scanned at 30 s intervals for each mouse. Moor LDI Image Review Software (Moor Instruments Ltd, Axminster, England) was then used to calculate perfusion within the region of interest for each image on the basis of colored histogram pixels, and results were averaged. Perfusion was expressed as ratio of perfusion of the ischemic to the non-ischemic limb.

### Flow Cytometry Analysis

Leukocyte profile was analyzed in blood samples obtained from tail vein according to a previously described procedure (22). In brief, erythrocytes were lysed and samples were stained with fluorochrome-conjugated antibodies specific for CD45, CD11b, Ly6G, and Ly6C. Analysis was performed with a Cytomics FC500 flow cytometer and data were analyzed by Kaluza software (both Beckman Coulter).

### Histological and Immunohistochemistry Analyses

Ischemic gastrocnemius muscles were fixed with 10% buffered formalin and cross-sections (5 μm thick) were prepared. Hematoxylin and eosin (H&E) staining was performed to identify and quantify the number of regenerated muscle fibers, that is, with central nuclei. To this end, a total of 3-5 randomly chosen fields within each cross-section for a given animal were captured using a Leica DMLB microscope connected to a Leica DC300F camera (Leica Microsystems, Wetzlar, Germany). Number of fibers with centrally nuclei was counted manually and expressed as percentage of total muscle fiber for a given field. Cross-sectional area (CSA) of regenerating muscle fibers was measured with Leica QWin software (Leica Microsystems, Wetzlar, Germany).

Muscle sections were also immunostained with a mouse monoclonal α-smooth muscle actin (α-SMA) antibody to identify arterioles as described earlier (10, 14). Arteriolar counts were performed manually, and arteriolar density was expressed as number of arterioles per field. All analyses were performed under light microscopy with Leica QWin software. For each parameter (number of regenerated fibers, CSA, and arteriolar density), results from each field for a given section were averaged.

### Real-Time Reverse Transcription-Polymerase Chain Reaction Analysis

The procedure was performed as previously described with slight modifications (10, 14). Ischemic and contralateral non-ischemic gastrocnemius muscle samples were collected immediately following euthanasia, snap-frozen in liquid nitrogen, and stored at −80°C. Approximatively 30 mg of muscle tissue were disrupted and homogenized in lysis buffer using the Tissue Lyser LT (Qiagen, Switzerland). Total RNA was then extracted with total RNeasy Fibrous Tissue Mini Kit (Qiagen, Switzerland) and reverse transcripted into cDNA using the PrimeScript™ RT Reagent Kit with gDNA eraser (TaKaRa Bio Inc., Japan) according to the manufacturer's protocols. Quantitative real-time PCR was performed in duplicates for each sample using a CFX96 Real-Time PCR detection system (Bio-Rad Laboratories Inc, Switzerland) with murine primers listed in Supplementary Table 1. For each sample, Ct of each target gene was normalized to the housekeeping gene 36B4 to determine ΔCT. Data were presented as relative expression using the formula 2^−Δ*CT*^ (23).

### Statistical Analysis

All data are expressed as mean ± standard deviation. All analyses were performed using GraphPad Prism 8 Prism software. The statistical test used for each experiment is indicated in the figure legends. *P* < 0.05 is considered statistically significant.

## Results

### Effect of wheel Exercise Before Experimental PAD

ApoE^−/−^ mice underwent 5 weeks of voluntary exercise training or remained sedentary before induction of experimental PAD.

**Figure 1:**
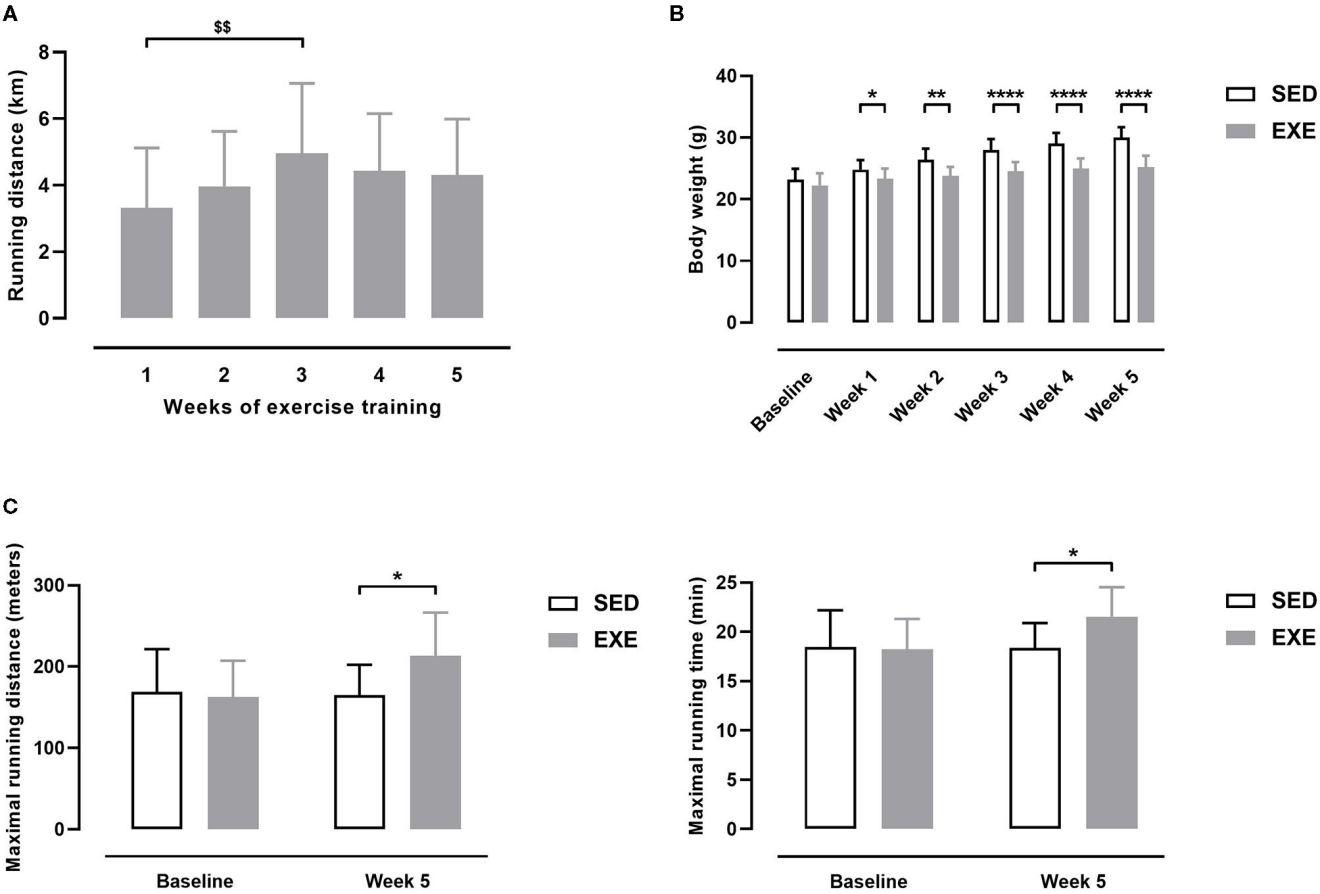
Effect of wheel exercise before induction of PAD in ApoE^−/−^ mice. **(A)** Daily distance ran each week by EXE mice (*n* = 11). **(B)** Body weights of EXE and SED mice (*n* = 11 animals per group). **(C)** Maximal running distance (left) and maximal running time (right) as measured on a graduated treadmill running test (*n* = 11 animals per group). Repeated measures one-way ANOVA with Tukey's multiple comparisons test in **(A)**: $$*p* < 0.01 vs. week 1. Unpaired *t*-test in **(B,C)**: *p < 0.05, **p < 0.01, ****p < 0.0001 vs. SED. Bar graphs represent mean ± SD.

Average daily running distance in EXE mice was 4.20 ± 1.59 km. As shown in Figure 1A, average daily running distance per week increased gradually the second week of training (3.97 ± 1.66 km), and the third one (4.96 ± 2.10 km, *p* < 0.01 vs. 3.32 ± 1.81 km the first week of training). Average daily distance slightly decreased the fourth week of training (4.44 ± 1.71 km), and remained stable the fifth one (4.31 ± 1.68 km) (Figure 1A).

At baseline, body weight (BW) did not significantly differ between SED and EXE groups (Figure 1B). EXE mice showed significantly lower BW than SED mice at each week of the 5-week study period (Figure 1B). Maximal running distance (MRD) and maximal running time (MRT) were similar between the two groups at baseline (Figure 1C). At the end of the 5-week study period, MRD and MRT were significantly improved in EXE mice compared to SED (+18 and +17% for MRD and MRT, respectively, *p* < 0.05; Figure 1C). Together, these data indicate that 5 weeks of voluntary wheel training induced specific physiological adaptations characteristics of aerobic exercise in our animals.

Ratio of tissue perfusion between right and left hindlimbs was 1.05 ± 0.07 in SED and 1.03 ± 0.04 in EXE mice at week 5 of the study period (non-significant), indicating similar perfusion between the right and left hindlimbs in both groups and that wheel exercise training had no effect on this parameter.

### Effect of Prior Wheel Exercise on Exercise Performance After Experimental PAD

**Figure 2:**
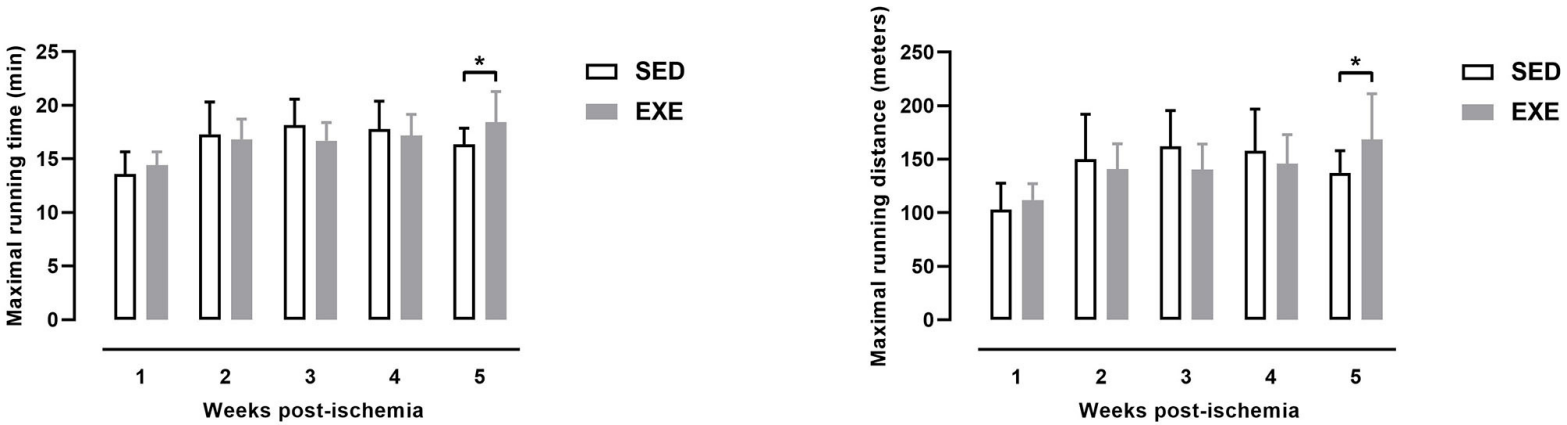
Effect of preventive exercise on physical performance in ApoE^−/−^ mice with PAD. Maximal running distance (left) and maximal running time (right) as measured on a graduated treadmill running test (*n* = 11 animals per group). Unpaired *t*-test: *p < 0.05 vs. SED. Bar graphs represent mean ± SD.

As shown in Figure 2, MRD and MRT were significantly higher in EXE mice than in SED at week 5 post-ischemia (MRD: 168.7 ± 42.5 vs. 137.3 ± 20.7 m, *p* < 0.05; MRT: 18.4 ± 2.9 min vs. 16.4 ± 1.5 min, *p* < 0.05). There were no significant differences in MRD and MRT at week 1, week 2, week 3, and week 4 post-ischemia between the two groups (Figure 2).

Similar to pre-ischemia period, EXE mice had significantly lower BW than SED mice each week post-ischemia (data not shown).

### Effect of Prior Wheel Exercise on Hindlimb Perfusion and Arteriogenesis After Experimental PAD

**Figure 3:**
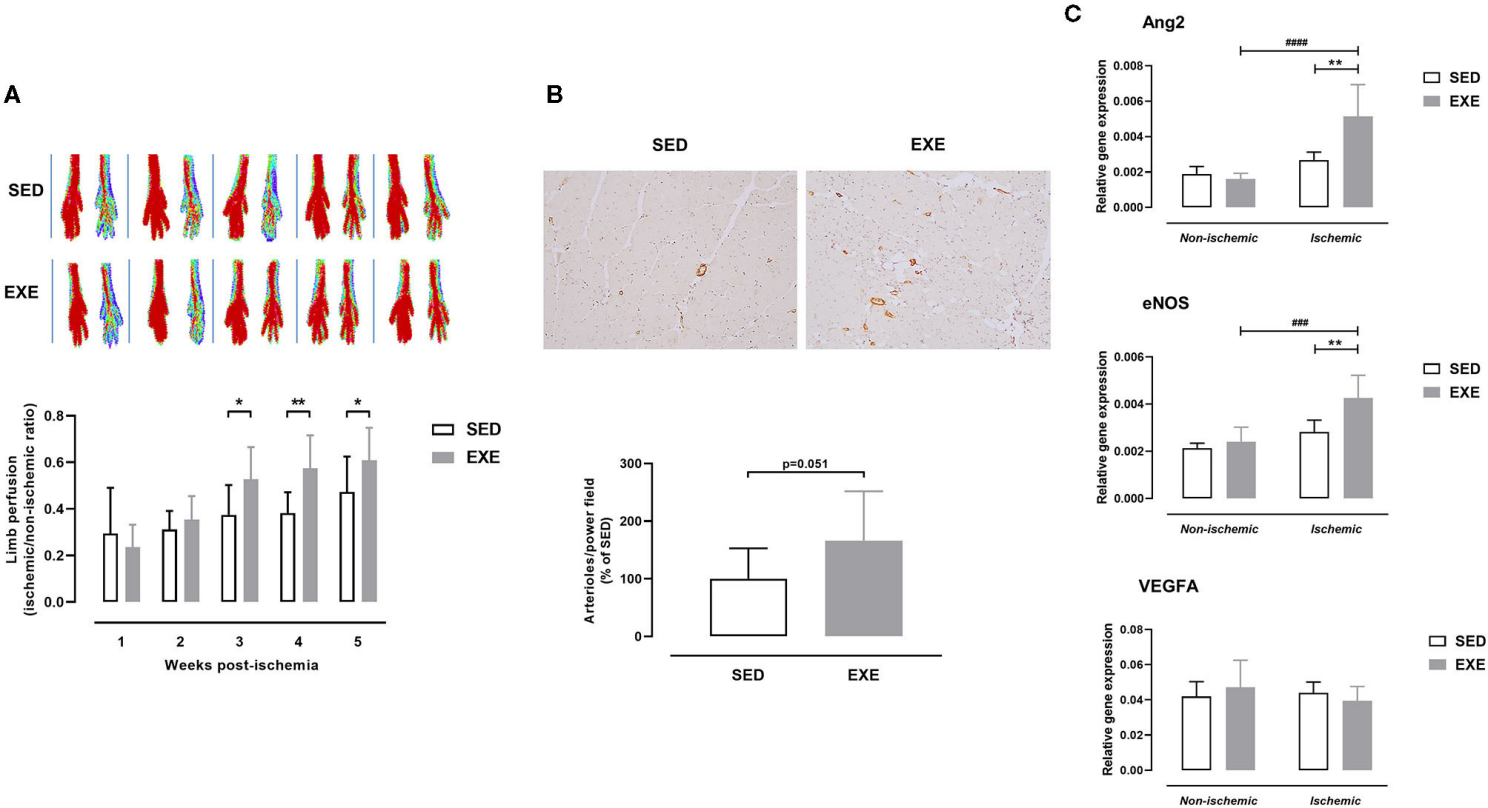
Effect of preventive exercise on ischemic hindlimb perfusion in ApoE^−/−^ mice with PAD. **(A)** Top: Representative laser Doppler images of ischemic (right) and contralateral non-ischemic (left) paws from week 1 to week 5 post-ischemia. Bottom: Ratio of perfusion in the ischemic hindlimb to the non-ischemic hindlimb (*n* = 11 animals per group). **(B)** Top, Representative images of ischemic gastrocnemius cross-sections immunostained with anti-α-SMA (in brown) at × 10 high-power field. Bottom: Quantification of arterioles number per high power field at week 5 post-ischemia (*n* = 10 animals per group). Data are expressed as the percentage of the mean value in SED. **(C)** Gene expression analysis by real-time RT-PCR of key genes involved in arteriogenesis (Ang2, eNOS, and VEGFA) in non-ischemic and ischemic gastrocnemius muscle of SED and EXE mice at week 5 post-ischemia (*n* = 5 animals per group). Unpaired *t*-test in **(A)** and **(B)**: *p < 0.05, **p < 0.01 vs. SED. Two-way ANOVA with Sidak's multiple comparisons test in **(C)**: ^###^*p* < 0.001, ^####^*p* < 0.0001 vs. respective non-ischemic; **p < 0.01 vs. SED. Bar graphs represent mean ± SD.

As shown in Figure 3A, perfusion of ischemic hindlimb did not significantly differ between EXE and SED mice at week 1 (0.24 ± 0.10 vs. 0.29 ± 0.20) and week 2 (0.35 ± 0.10 vs. 0.31 ± 0.08) post-ischemia. However, EXE mice showed improved ischemic hindlimb perfusion compared to SED at week 3 (0.53 ± 0.14 vs. 0.38 ± 0.13, *p* < 0.05), week 4 (0.58 ± 0.14 vs. 0.38 ± 0.09, *p* < 0.01), and week 5 (0.61 ± 0.14 vs. 0.47 ± 0.15, *p* < 0.05) post-ischemia (Figure 3A). Histological analysis showed that number of arterioles per high-power field was also increased in ischemic muscle of EXE compared to SED at week 5 post-ischemia (+66%, *p* = 0.051; Figure 3B). Quantitative real-time RT-PCR analysis revealed that the expression of pro-arteriogenic genes Ang2 and eNOS, but not VEGFA, were significantly upregulated in ischemic muscle of EXE mice as compared to SED (*p* < 0.01, Figure 3C).

**Figure 4:**
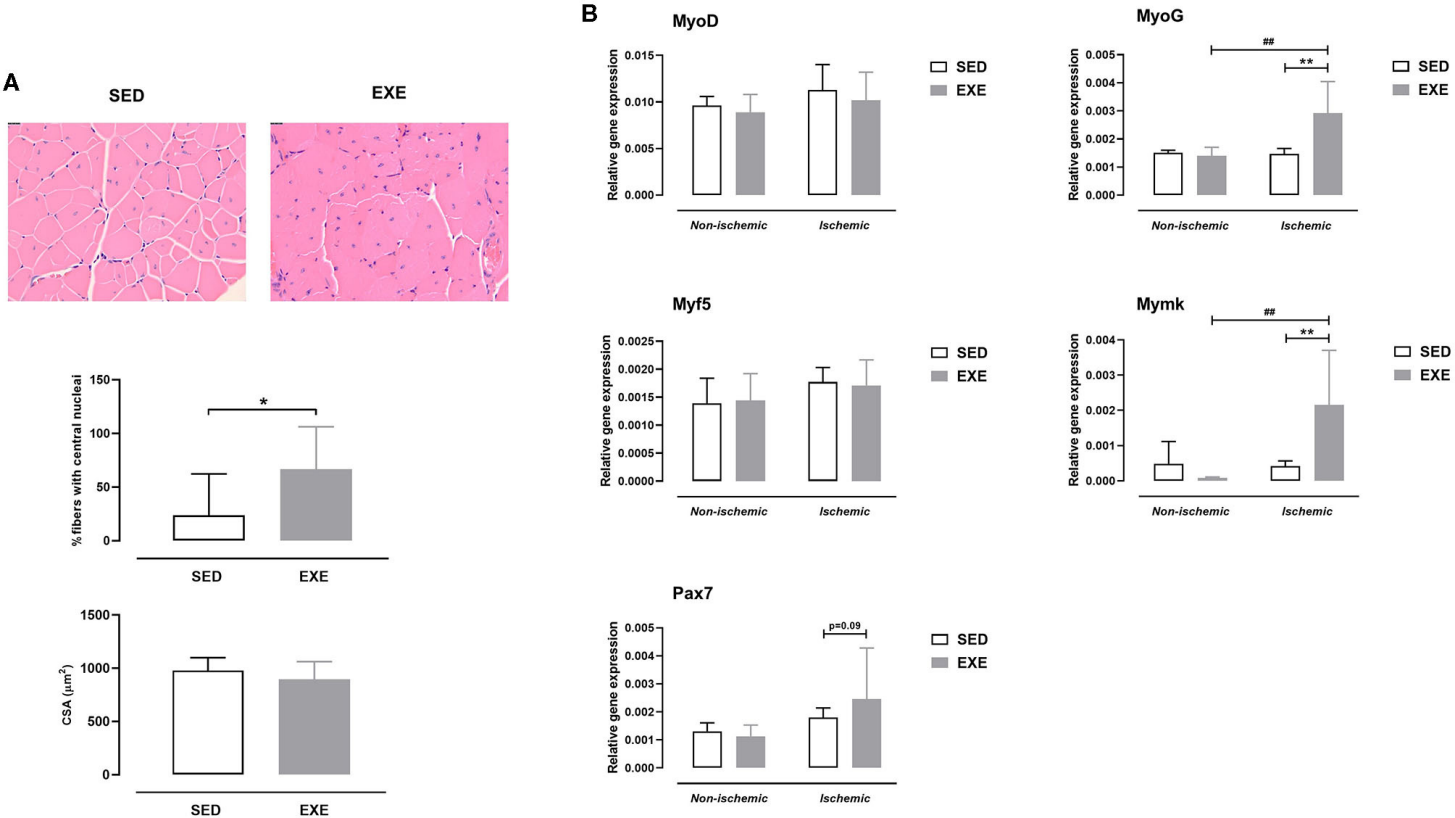
Effect of preventive exercise on ischemic hindlimb muscle regeneration in ApoE^−/−^ mice with PAD. **(A)** Top: Representative images of ischemic gastrocnemius cross-sections stained with H&E at × 20 high-power field. Middle: Quantification of centro-nucleated moyfibers at week 5 post-ischemia (*n* = 7-10 animals per group). Bottom: Quantification of cross-sectional area (CSA) of regenerating ischemic gastrocnemius myofibers (*n* = 3-9 animals per group). **(B)** Gene expression analysis by real-time RT-PCR of key genes involved in muscle regeneration (MyoD, MyoG, Myf5, Mymk, and Pax7) in non-ischemic and ischemic gastrocnemius muscles of SED and EXE mice at week 5 post-ischemia (*n* = 5 animals per group). Unpaired *t*-test in **(A)**: *p < 0.05 vs. SED. Two-way ANOVA with Sidak's multiple comparisons test in **(B)**: ##*p* < 0.01 vs. respective non-ischemic; **p < 0.01 vs. SED. Bar graphs represent mean ± SD.

As shown in Figure 4A, there was a significantly higher number of regenerating myofibers in the ischemic muscle of EXE than in SED (66.8 ± 39.4 vs. 23.9 ± 38.4%, *p* < 0.05). In agreement with the histological findings, expression of myogenesis-related genes MyoG and Mymk was also upregulated in ischemic muscle of EXE mice (*p* < 0.01 vs. SED; Figure 4B). In addition, Pax7 gene expression tended to increase (*p* = 0.09) in ischemic muscle of EXE compared to SED (Figure 4B). No significant difference in CSA of regenerating myofibers was found between the two groups (Figure 4A).

### Effect of Prior Wheel Exercise on Hindlimb Muscle Macrophage Infiltration and Profile After Experimental PAD

**Figure 5:**
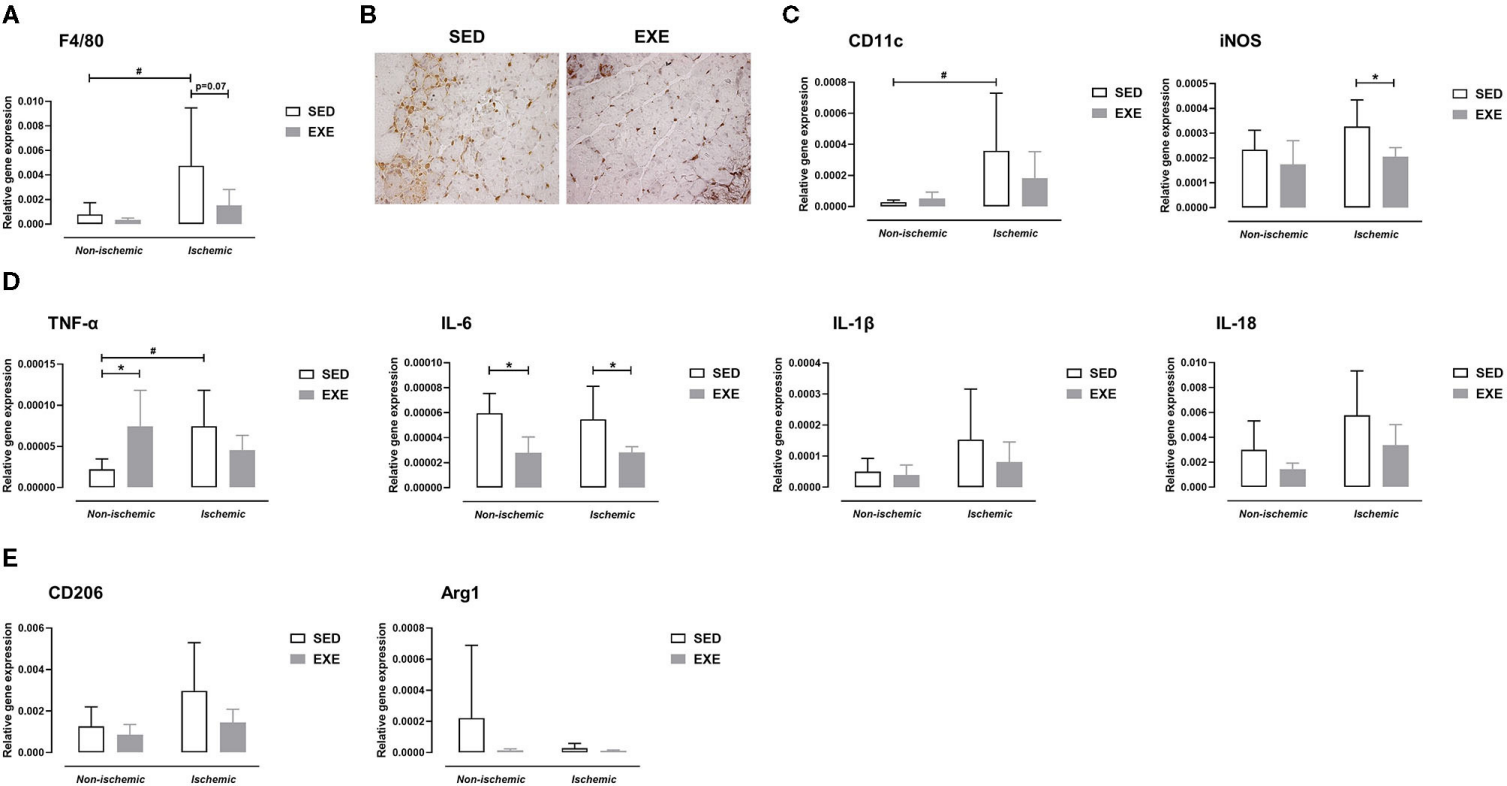
Effect of preventive exercise on hindlimb muscle inflammation of ApoE^−/−^ mice 1 week after PAD. **(A)** Gene expression analysis by real-time RT-PCR of the F4/80 macrophage marker in non-ischemic and ischemic gastrocnemius muscles of SED and EXE mice (*n* = 6 animals per group). **(B)** Representative images of ischemic gastrocnemius cross-sections stained with anti-Mac-2 (in brown) at × 20 high-power field. **(C-E)** Gene expression analysis by real-time RT-PCR of pro-inflammatory M1 macrophage markers **(C)**, M1-related pro-inflammatory cytokines **(D)**, and of anti-inflammatory M2 macrophage markers **(E)** in non-ischemic and ischemic gastrocnemius muscles of SED and EXE mice (*n* = 4-6 animals per group). Two-way ANOVA with Sidak's multiple comparisons test in **(A,C-E)**: #*p* < 0.05 vs. respective non-ischemic; *p < 0.05 vs. SED. Bar graphs represent mean ± SD.

At week 1 post-ischemia, gene expression analysis revealed upregulation of the macrophage marker F4/80 in the ischemic muscle of SED mice (*p* < 0.05 vs. the non-ischemic muscle), but not in that of EXE mice (Figure 5A). F4/80 gene expression tended to be reduced in the ischemic muscle of EXE when compared to that of SED (*p* = 0.07; Figure 5A). In accord with the results of real-time RT-PCR, immunostaining against the macrophage marker Mac-2 revealed less macrophage infiltration in the ischemic muscle of EXE than in that of SED (Figure 5B). We next characterized the phenotype of macrophages using specific markers for M1 and M2 macrophages using real-time RT-PCR. As we previously reported (14), SED mice exhibited significant increase in M1 marker CD11c expression in the ischemic muscle (*p* < 0.05 vs. non-ischemic muscle), while no significant increase was observed in EXE mice (Figure 5C). Gene expression of iNOS, a second specific marker for M1 macrophage, was significantly reduced in ischemic muscle of EXE mice in comparison with SED (*p* < 0.05; Figure 5C). Because M1 macrophages are associated with production of pro-inflammatory cytokines, we next determined gene expression of TNF-α, IL-6, IL-1β, and IL-18. In line with CD11c, TNF-α expression was also significantly increased in the ischemic muscle of SED (*p* < 0.05 vs. non-ischemic; Figure 5D). This increase was inhibited in EXE mice (Figure 5D). Surprisingly, a significant increase in TNF-α expression (*p* < 0.05) was observed in the non-ischemic muscle of EXE mice when compared to that of EXE (Figure 5D). Furthermore, compared to SED, IL-6 expression was significantly reduced in EXE both in the ischemic and non-ischemic muscle (*p* < 0.05 vs. SED; Figure 5D). Finally, although not significant, there was a trend toward higher IL-1β and IL-18 expression in the ischemic muscle of SED mice compared to the non-ischemic muscle, which was not reported in EXE mice (Figure 5D). Analysis of M2 macrophage markers CD206 and Arg1 revealed no significant difference between the two groups (Figure 5E).

**Figure 6:**
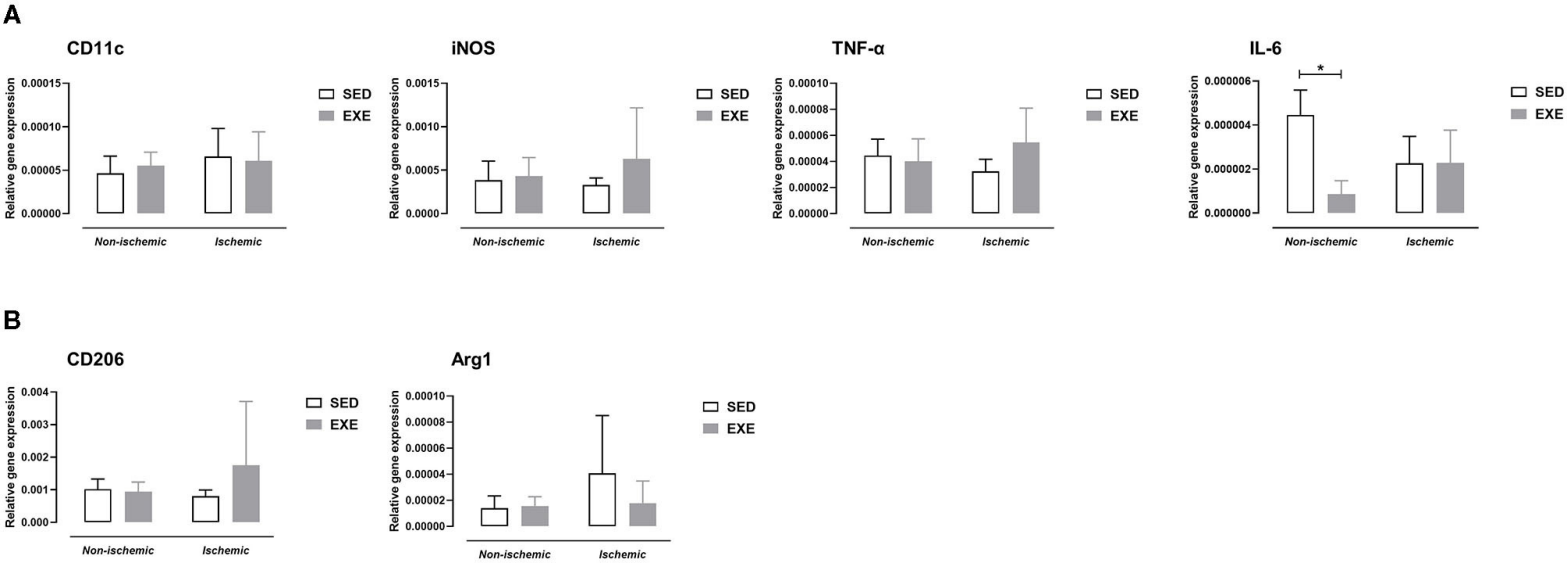
Effect of preventive exercise on hindlimb muscle inflammation of ApoE^−/−^ mice 5 weeks after PAD. Gene expression analysis by real-time RT-PCR of pro-inflammatory M1 macrophage markers and cytokines **(A)**, and of anti-inflammatory M2 macrophage markers **(B)** in non-ischemic and ischemic gastrocnemius muscles of SED and EXE mice (*n* = 4-6 animals per group). Two-way ANOVA with Sidak's multiple comparisons test in **(A,B)**: *p < 0.05 versus SED. Bar graphs represent mean ± SD.

At week 5 post-ischemia, neither M1 macrophage markers, nor M2 macrophages markers expression were significantly different between EXE and SED (Figure 6).

### Effect of Prior Wheel Exercise on Circulating Monocyte Subtypes Profile After Experimental PAD

**Figure 7:**
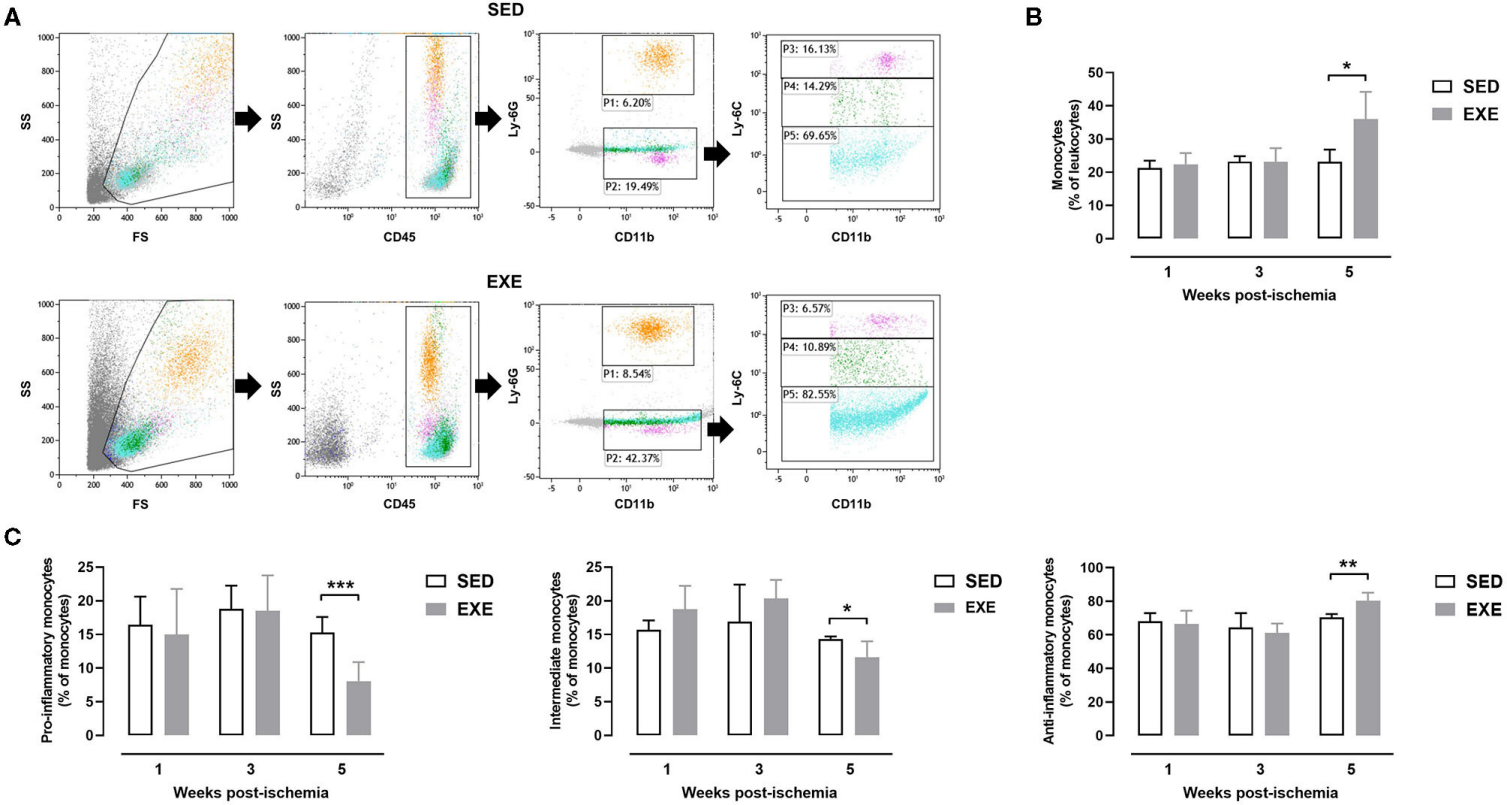
Effect of preventive exercise on circulating monocytes of ApoE^−/−^ mice with PAD. **(A)** Example of gating strategies to identify monocyte subsets in SED and EXE mice. First, viable cells were gated on Forward Scatter (FS)/Side Scatter (SS). Next, a CD45/SS plot was created within the viable cells to identify leukocytes (CD45^+^ cells). Granulocytes were identified as CD11^+^ Ly6G^+^ cells (P1), while monocytes were identified as CD11^+^ Ly6G^−^ cells (P2). Monocytes were further gated for expression of Ly6C, after which three distinct subpopulations of monocytes were categorized as follows: classical proinflammatory monocytes (CD11^+^ Ly6G^−^ Ly6C^hi^), intermediate monocytes (CD11^+^ Ly6G^−^ Ly6C^int^) and non-classical anti-inflammatory monocytes (CD11^+^ Ly6G^−^ Ly6C^low^). **(C)** The percentage of monocytes within the leukocyte population (**B)** and of each monocyte subtype within the monocyte population were compared in SED and EXE mice at 1 week, week 3, and week 5 post-ischemia (*n* = 4 to 11 animals per group). Unpaired *t*-test in **(B,C)**: *p < 0.05, **p < 0.01, and ***p < 0.001 vs. SED. Bar graphs represent mean ± SD.

Flow cytometry analysis of peripheral blood showed that the proportion of monocytes was significantly higher in EXE than SED mice at week 5 post-ischemia (36.0 ± 8.2 vs. 23.1 ± 3.7%, *p* < 0.05; Figures 7A,B). Further analysis of monocyte subtypes revealed a significant decrease in the proportion of pro-inflammatory monocytes in EXE as compared to SED mice at week 5 post-ischemia (8.1 ± 2.8 vs. 15.3 ± 2.3%, *p* < 0.001; Figures 7A,C). A significant reduction of intermediate monocytes proportion was also observed (11.6 ± 2.4 vs. 14.3 ± 0.4%, *p* < 0.05; Figures 7A,C). On the contrary, the proportion of anti-inflammatory monocytes significantly increased in EXE (80.3 ± 4.7 vs. 70.4 ± 2.0% in SED, *p* < 0.01, Figures 7A,C). Neither the proportion of monocytes, nor that of monocyte subtypes was significantly different between the two groups at week 1 and week 3 post-ischemia (Figure 7C). The proportion of granulocytes did not significantly differ between the two groups at any time point post-ischemia (data not shown).

## Discussion

The main finding of the present study is that 5 weeks of voluntary wheel exercise initiated before PAD improved running capacity post-PAD in ApoE^−/−^ mice. In addition, we demonstrated that exercise training prior to PAD stimulated ischemic limb arteriogenesis/blood perfusion, and modulated phenotype polarization status of circulating monocytes and muscle macrophages.

The role of physical activity and exercise training in the primary and secondary prevention of cardiovascular disorders has been extensively demonstrated (2, 3). In animal models, we and others have shown that aerobic exercise (voluntary running, forced running, and swimming) prevents the development of atherosclerotic plaques and slows their progression into advanced or severe stage (24–26). Swimming and treadmill running also protect against experimental acute myocardial infarction (27) and stroke (28). Furthermore, it has been shown that 5 weeks of voluntary running preserves motor nerve and myofiber structure and function in a mouse model of hindlimb ischemia-reperfusion (29). However, few studies have investigated the preventive (or preconditioning) effect of exercise in animal models of cardiovascular diseases. For instance, de Waard and Duncker have demonstrated that 2 weeks of voluntary wheel running before permanent ligation of the left anterior descending coronary artery (mouse model of myocardial infarction) decreased post-myocardial infarction mortality and improved cardiac function in C57BL/6 mice (30). More recently, Naderi et al. demonstrated that 4 weeks of treadmill running preconditioning diminished infarct volume, and improved neurological deficits after permanent middle cerebral artery occlusion (experimental model of stroke) in ovariectomized mice (31). Our investigation is the first one to our knowledge having examined the preventive effect of exercise in the setting of PAD, and demonstrating that exercise is an effective preventive therapy to delay PAD-related decreased running capacity.

We next wondered whether preventive exercise could stimulate post-ischemia arteriogenic gene expression, thereby increasing vascularization and limb blood flow. There are controversies in the literature about the effects of therapeutic exercise training on hemodynamic parameters in mouse model of hindlimb ischemia, with some studies reporting improved arteriogenesis and perfusion recovery following exercise training (32, 33), while others showing no effect (9, 10). The increased gene expression of Ang2 and eNOS and number of arterioles in the ischemic muscle, as well as the enhanced perfusion in the ischemic hindlimb in exercised mice in comparison with sedentary ones confirmed our hypothesis. In agreement with our findings, the elegant study from Schirmer et al. demonstrating increased perfusion restoration in ApoE^−/−^ mice exercising for 3 weeks (voluntary running wheel) before right femoral artery ligation (34). The authors also reported increased number of collateral arteries in the ischemic adductor muscle of exercising mice (34). Our investigation however provide novel insights as we showed enhanced perfusion with preconditioning exercise late after peripheral ischemia (i.e., at 3 weeks, 4 weeks, and 5 weeks post-ischemia) while Schirmer's study observed such effect earlier (i.e., 1 week post-ischemia) (34). Our result is of particular interest from a clinical point of view for the prevention of long-term (chronic) disease such as atherosclerotic PAD. Moreover, we showed evidence of accelerated muscle regeneration late after ischemia, as demonstrated by significant increased number of myofibers with centrally located nuclei and gene expression of promyogenic factors MyoG and Mymk, characteristic of improved muscle recovery. It is noteworthy that Schirmer et al. did not measure endurance capacity in their mice contrary to our study showing that the preventive exercise-induced augmentation of ischemic limb blood flow translated into functional outcome improvement. Taken together, our findings and those from Schirmer et al. collectively indicate that exercise is able to improve lower limb hemodynamic after ischemia even if exercise is stopped before ischemia.

At the cellular and molecular level, the present study shows that pre-exercise prevents increased gene expression levels of F4/80 and M1-like macrophage marker CD11c as well as pro-inflammatory cytokine TNF-α in the ischemic muscle at week 1 post-PAD. We also observed decreased Mac-2 positive cells (qualitative analysis) as well as decreased M1 marker iNOS and IL-6 gene expression in the ischemic muscle in response to exercise. It has been previously proven that reducing macrophages in the ischemic skeletal muscle of mice enhances vascularization, promotes tissue regeneration, and/or improves recovery of hindlimb use (35–37). In the same line, a previous study reported that reducing levels of pro-inflammatory cytokines TNF-α, IL-6, and IL-1 in ischemic muscle is associated with better treadmill running performance in mice (37). A reduction in TNF-α mRNA expression in ischemic gastrocnemius muscle has also been linked with a higher artery density and an improvement in perfusion recovery 1 week after experimental PAD in mice after treatment with hydrogen molecules (13). We also previously reported that inhibiting ischemic muscle inflammation at week 1 post-PAD, especially pro-inflammatory M1 activation state, resulted in improved endurance capacity (14). Herein, we show for the first time that exercise is able to modulate muscle inflammation and M1 polarization-associated genes in PAD. It should be emphasized that preventive exercise had no effect on M1 markers gene expression at week 5 post-PAD. This is not surprising since muscle inflammation is not present anymore at this time point post-PAD as we previously reported (14). Besides a reduction in M1 macrophages, one could also expect an increased number of M2 macrophages with exercise training on the basis of previous studies showing that exercise can polarize macrophages toward the M2 phenotype in skeletal muscle (19, 21). In the present study, we did not observe any impact of preventive exercise on ischemic muscle M2 macrophage gene expression at either 1 or 5 week post-PAD. The lack of changes in M2 macrophage phenotype might explain why VEGFA gene expression in ischemic muscle was not modulated with preventive exercise, since M2 macrophages are a major source of VEGF production (11). In agreement with our study, a reduction in M1 macrophages without affecting M2 macrophages has been also observed in gastrocnemius muscle from an animal model of Duchenne muscular dystrophy after 4 weeks of low intensity training (38). Taken together, our data indicate that exercise prior to PAD promotes arteriogenesis and muscle regeneration by inhibiting inflammation rather than an anti-inflammatory effect in our mouse model.

An important novel finding of the current study is that the proportion of circulating pro-inflammatory monocytes decreased while that of anti-inflammatory monocytes increased following preventive exercise at 5 weeks post-PAD, indicating a polarization shift toward an anti-inflammatory phenotype. Surprisingly, no effect of exercise on circulating monocytes phenotype could be observed at 1 week post-PAD although a decreased in muscle M1 macrophages occurred at this time point as described above. These data suggest that the effect of prior exercise on muscle macrophages does not reflect at the systemic level. It could be therefore hypothesized that resident macrophages within the skeletal ischemic muscle rather than the newly recruited monocytes-derived macrophages are responsible for arteriogenesis following pre-exercise in our mouse model. The study by Khmelewski et al. showing that tissue resident macrophages play a dominant role in arteriogenesis independently of circulating monocytes supports our hypothesis (39). Further study is required, however, to better understand the role of circulating monocyte subtypes in PAD pathophysiology.

In summary, we show that exercise initiated before PAD ameliorates endurance performance and hindlimb perfusion in mice. Mechanistically, we propose that the inhibition of M1 macrophage polarization and inflammation in the early stage of muscle ischemia may underlie the observed beneficial effect of preventive exercise.

Our findings have potential clinical application. Initiating regular physical activity early in adult life would attenuate disease severity in individuals who may develop PAD during their life. In addition, this study further reinforced the importance of physical activity for primary prevention of cardiovascular disease, and especially of PAD.

## Data Availability Statement

The original contributions presented in the study are included in the article/supplementary material, further inquiries can be directed to the corresponding author/s.

## Ethics Statement

The animal study was reviewed and approved by the ethical committee of the canton de Vaud.

## Author Contributions

MP and LM conceived/designed the study, interpreted data, and wrote the manuscript. MP and KB performed experiments. MP, KB, and LM analyzed data. All authors read and approved the final version of this manuscript.

## Conflict of Interest

The authors declare that the research was conducted in the absence of any commercial or financial relationships that could be construed as a potential conflict of interest.

## Publisher's Note

All claims expressed in this article are solely those of the authors and do not necessarily represent those of their affiliated organizations, or those of the publisher, the editors and the reviewers. Any product that may be evaluated in this article, or claim that may be made by its manufacturer, is not guaranteed or endorsed by the publisher.
