# Supplementary material for: Exercise Prior to Lower Extremity Peripheral Artery Disease Improves Endurance Capacity and Hindlimb Blood Flow by Inhibiting Muscle Inflammation
Source: Front Cardiovasc Med. 2021 Aug 4;8:706491. doi: 10.3389/fcvm.2021.706491 (PMC8371529; doi:10.3389/fcvm.2021.706491)
Supplement: Supplementary file 1 [file Table_1.docx]

**Supplementary Table 1.** Mouse primer sequences used for quantitative real-time PCR.

| **Gene** | **Forward sequence** | **Reverse sequence** | **Code** | **Amplicon** |
| --- | --- | --- | --- | --- |
| 36B4 | 5’-ATGGGTACAAGCGCGTCCTG-3’ | 5’-GCCTTGACCTTTTCAGTAAG-3’ | NM_007475.5 | 72 |
| ANG2 | 5’-GCATGTGGTCCTTCCAACTT-3’ | 5’-TGGTGTCTCTCAGTGCCTTG-3’ | NM_007426 | 215 |
| eNOS | 5’-GACCCTCACCGCTACAACAT-3’ | 5’-CTGGCC TTCCGCTCATTTTC-3’ | NM_008713 | 208 |
| VEGFA | 5’-TGCACCCACGACAGAAGG-3’ | 5’-GCACACAGGACGGCTTGA-3’ | NM_009505 | 155 |
| MyoD | 5’-GCTGCCTTCTACGCACCTG-3’ | 5’- GCCGCTGTAATCCATCATGC-3’ | NM_010866.2 | 119 |
| MyoG | 5’-GCAATGCACTGGAGTTCG-3’ | 5’-GGTCTGGGTTCCCTGTTCTGTT-3’ | NM_031189.2 | 99 |
| Myf5 | 5’-CCTGTCTGGTCCCGAAAGAAC-3’ | 5’- GACGTGATCCGATCCACAATG-3’ | NM_008656.5 | 130 |
| Mymk | 5’-CCTGCTGTCTCTCCCAAG-3’ | 5’-AGAACCAGTGGGTCCCTAA-3’ | NM_025376.3 | 133 |
| Pax7 | 5’-CTCAGTGAGTTCGATTAGCCG-3’ | 5’-AGACGGTTCCCTTTGTCGC-3’ | NM_011039.2 | 143 |
| F4/80 | 5’-TGGGATGTACAGATGGGGGA-3’ | 5’- CCTGGGCCTTGAAAGTTGGT-3’ | X93328.1 | 188 |
| CD11c | 5’-ACACAGTGTGCTCCAGTATGA-3’ | 5’-GCCCAGGGATATGTTCACAGC-3’ | NM_021334.2 | 155 |
| iNOS | 5’-CCAAGCCCTCACCTACTTCC-3’ | 5’- CTCTGAGGGCTGACACAAGG -3’ | NM_010927.3 | 126 |
| TNF-α | 5’-TAGCCAGGAGGGAGAACAGAAAC-3’ | 5’-CCAGTGAGTGAAAGGGACAGAAC-3’ | Nm_001278601.1 | 98 |
| IL-6 | 5’-GATGCTACCAAACTGGATATAATC-3’ | 5’- GGTCCTTAGCCACTCCTTGTGTG-3’ | NM_001314054.1 | 210 |
| IL-1β | 5’-TGAAGTTGACGGACCCCAAAA-3’ | 5’-TGATGTGCTGCTGTGAGATT-3’ | Nm_008361.1 | 100 |
| IL-18 | 5’-ACGTGTTCCAGGACACAACA-3’ | 5’- ACAAACCCTCCCCACCTAAC-3’ | NM_008360 | 180 |
| CD206 | 5’-CATGGATGTTGATGGCTACTGGAG-3’ | 5’-GTCTGTTCTGACTCTGGACACTTG-3’ | NM_008625.2 | 131 |
| Arg1 | 5’-CTCCAAGCCAAAGTCCTTAGAG-3’ | 5’-AGGAGGTGTCATTAGGGACATC-3’ | NM_0074482 | 184 |
